# Divergent structural brain abnormalities between different genetic subtypes of children with Prader–Willi syndrome

**DOI:** 10.1186/1866-1955-5-31

**Published:** 2013-10-22

**Authors:** Akvile Lukoshe, Tonya White, Marcus N Schmidt, Aad van der Lugt, Anita C Hokken-Koelega

**Affiliations:** 1 Dutch Growth Research Foundation, Postbus 23068, Rotterdam 3001, KB, The Netherlands; 2 Department of Pediatrics, Erasmus Medical Center Rotterdam – Sophia Children’s Hospital Rotterdam, Postbus 2060, Rotterdam 3000, CB, The Netherlands; 3 Department of Child and Adolescent Psychiatry, Erasmus Medical Center Rotterdam – Sophia Children’s Hospital, Postbus 2060, Rotterdam 3000 CB, The Netherlands; 4 Department of Radiology, Erasmus Medical Center Rotterdam, Postbus 2040, Rotterdam 3000 CA, The Netherlands

**Keywords:** Prader–Willi syndrome, Neurodevelopmental disorder, Chromosome 15q11-q13, Structural MRI

## Abstract

**Background:**

Prader–Willi syndrome (PWS) is a complex neurogenetic disorder with symptoms that indicate not only hypothalamic, but also a global, central nervous system (CNS) dysfunction. However, little is known about developmental differences in brain structure in children with PWS. Thus, our aim was to investigate global brain morphology in children with PWS, including the comparison between different genetic subtypes of PWS. In addition, we performed exploratory cortical and subcortical focal analyses.

**Methods:**

High resolution structural magnetic resonance images were acquired in 20 children with genetically confirmed PWS (11 children carrying a deletion (DEL), 9 children with maternal uniparental disomy (mUPD)), and compared with 11 age- and gender-matched typically developing siblings as controls. Brain morphology measures were obtained using the FreeSurfer software suite.

**Results:**

Both children with DEL and mUPD showed smaller brainstem volume, and a trend towards smaller cortical surface area and white matter volume. Children with mUPD had enlarged lateral ventricles and larger cortical cerebrospinal fluid (CSF) volume. Further, a trend towards increased cortical thickness was found in children with mUPD. Children with DEL had a smaller cerebellum, and smaller cortical and subcortical grey matter volumes. Focal analyses revealed smaller white matter volumes in left superior and bilateral inferior frontal gyri, right cingulate cortex, and bilateral precuneus areas associated with the default mode network (DMN) in children with mUPD.

**Conclusions:**

Children with PWS show signs of impaired brain growth. Those with mUPD show signs of early brain atrophy. In contrast, children with DEL show signs of fundamentally arrested, although not deviant brain development and presented few signs of cortical atrophy. Our results of global brain measurements suggest divergent neurodevelopmental patterns in children with DEL and mUPD.

## Background

Prader–Willi syndrome (PWS) is a rare and poorly understood neurodevelopmental disorder that affects 1 in 15,000 to 20,000 live births [1]. PWS is characterized by pre- and postnatal hypotonia, endocrine problems, hyperphagia, temper tantrums and repetitive behavior, skin picking, dysmorphic facial features, high pain threshold, and developmental delays [2]. In addition, individuals with PWS carry a high risk of psychiatric illness, such as psychotic, obsessive-compulsive disorder (OCD), and autism spectrum disorders (ASD).

The underlying cause of PWS is the loss of function of paternally expressed genes on the long arm of chromosome 15q11-q13 due to a *de novo* deletion (DEL, approximately 70% of the cases [3]), maternal uniparental disomy (mUPD, 25% of the cases [4]), and unbalanced translocation or imprinting center defects (<5% of cases [5]).

Lifetime prevalence of psychotic illness in individuals with PWS is reported to be up to 60% in individuals with mUPD and up to 20% in individuals with DEL [1], which is at least 18 times higher than that in the general population [6]. In addition, the mUPD patients are more likely to have a more severe course of the psychiatric illness, a higher frequency of relapse, and a poorer response to medication [7]. The underlying neurobiology that places them at-risk is yet unknown. Recently, copy-number variations (CNVs) at the 15q11.2 locus were found to be associated with schizophrenia and related psychosis [8], which suggests common genetic pathways between schizophrenia, psychotic disorders, and PWS.

In addition, individuals with PWS have a higher prevalence of ASD traits, such as impaired social functioning, rigidity, resistance to change, and repetitive, stereotypic behavior [9]. These autistic traits are more common in individuals with mUPD than in those with DEL (40% versus 20%, respectively). The autistic symptoms found in PWS are between 20 to 40 times higher than the 1% reported in the general population [10]. Chromosomal rearrangements of 15q11-13 locus have been found in patients with autism [11], suggesting that defective 15q11-13 genes might underlie the ASD traits in individuals with PWS. Further, recent findings indicate a plausible common neurodevelopmental pathway of autism, schizophrenia, and bipolar disorder, as family history of schizophrenia and bipolar disorder increased risk of autism in other family members [12], and several CNVs in genome-wide association studies (GWASs) were associated with developing either of these disorders [13].

Key symptoms indicate that the central nervous system (CNS) is adversely affected in PWS. To date, only two quantitative structural magnetic resonance imaging (MRI) studies have been performed in adults with PWS, which reported smaller grey matter volumes in the frontal, temporal, and parietal [14,15] lobes, and smaller white matter volumes in the frontal and temporal cortices, brainstem, and cerebellum [15]. An MRI qualitative study reported enlarged ventricles, decreased parieto-occipital lobar volume, and sylvian fissure abnormalities in adults with PWS [16]. However, no quantitative structural or functional MRI studies have been performed in children with PWS, and there are no studies evaluating differences between children with DEL and mUPD. Since the DEL and mUPD subtypes of PWS differ in their risk of developing severe psychiatric disorders, a better understanding of the neurobiology of these subtypes will shed light on at-risk states.

In the current study, we utilized high resolution MRI to investigate the brain morphology in children with two subtypes of PWS (DEL and mUPD) in order to gain insights into the brain structure of young children with PWS as compared with their healthy siblings. We hypothesized that children with PWS, when compared with healthy siblings, will show global deficits that will be reminiscent of those reported in the adults with PWS. Specifically, we expected smaller overall grey and white matter volume and larger lateral ventricular volume in children with PWS when compared with healthy controls. Since clinical data show that children with mUPD have greater social and cognitive deficits than children with DEL, we expected that the brain differences would be more pronounced in children with mUPD than in those with DEL. Furthermore, since children with mUPD show an increased risk of psychiatric disorders over children with DEL, we were also interested in brain differences which may indicate an increased risk of developing a severe psychiatric disorder.

## Methods

The study population consisted of 25 children with PWS who participated in the Dutch PWS Cohort Study [17]. Patients fulfilled the following criteria: 1) genetically confirmed PWS subtype; 2) age 6 to 18 years old; and 3) no neurological or psychiatric history.

Eleven age- and gender-matched, typically developing siblings were included as a control group fulfilling the following inclusion criteria: 1) age 6 to 18 years old; and 2) no neurological or psychiatric history.

This study was approved by the Medical Ethical Committee of the Erasmus Medical Center Rotterdam, Rotterdam, The Netherlands. Written informed consent was obtained in all cases from the caregivers and children older than 12 years, and assent of children younger than 12 years.

### Intelligence assessment

To assess intelligence of children, a short form of four subtests (vocabulary, similarities (verbal IQ subtests), block design, and picture arrangement (performance IQ subtests)) of the Wechsler Intelligence Scale for Children-Revised (WISC-R), Dutch version, was used [18]. Total IQ score was calculated according to an equation based on the Dutch outpatient population reference (total IQ = 45.3 + 2.91 × vocabulary standard score + 2.50 × block design standard score), as has been used in other studies [19].

### Procedure

Prior to the MRI scan, all children were introduced to a mock scanner and all successfully completed the mock scanner protocol [20]. One of the caregivers opted to stay in the room with the MRI scanner, to remain close to their child.

### MRI acquisition

Imaging was performed on a 3T GE 750 Discovery MRI scanner (General Electric, Milwaukee, WI, USA), using a dedicated 8-channel head coil. Following 3-plane localizing and coil intensity calibration scans, a high resolution T1-weighted inversion recovery fast spoiled gradient recalled (IR FSPGR) sequence was obtained with the following parameters: TR = 10.3 ms, TE = 4.2 ms, TI = 350 ms, NEX = 1, flip angle = 16°, readout bandwidth = 20.8 kHz, matrix 256 × 256, imaging acceleration factor of 2, and an isotropic resolution of 0.9 × 0.9 × 0.9 mm^3^ (duration: 5 minutes 40 seconds). All MRI images were reviewed by a qualified radiologist (AvdL) within 2 weeks after the MRI acquisition. No gross brain abnormalities were identified.

### Data preprocessing and segmentation

Five children with PWS were excluded from the MRI analysis due to motion artifacts, and failure of proper pial and white surface reconstruction by FreeSurfer (Athinoula A Martinos Center for Biomedical Imaging, Massachusetts General Hospital, Boston, MA, USA; http://surfer.nmr.mgh.harvard.edu), leaving 20 eligible patients for analysis (11 children with DEL, 9 children with mUPD).

Cortical reconstruction and volumetric segmentation was performed with the FreeSurfer 5.3 image analysis suite. The technical details of these procedures are described elsewhere [21-23]. Briefly, the processing included removal of non-brain tissue [22,24], automated Talairach transformation, segmentation of the subcortical white matter and deep grey matter volume structures [23,24], intensity normalization [25], and automated topology correction [26]. Parcellation of the cerebral cortex into units was performed based on gyral and sulcal structure [27]. Cortical thickness was calculated as the closest distance from the grey/white boundary to the grey/cerebrospinal fluid (CSF) boundary at each vertex on the tessellated surface [28]. Procedures for the measurement of cortical thickness have been validated against histological analysis [29]. FreeSurfer morphometric procedures have been demonstrated to show good test-retest reliability across scanner manufacturers and across field strengths [30]. All FreeSurfer outputs were manually reviewed by an author blinded to the patient data (AL), and manual edits were performed where needed to improve the white and pial surface reconstruction.

### Statistical analysis

Results of cerebral cortex parcellation, based on gyral and sulcal structure, were exported to SPSS (version 20, IBM Corporation, Armonk, NY, USA) for appropriate statistical analyses. Nonparametric Kruskal–Wallis tests were performed for both global brain morphology measures and exploratory subcortical grey and white matter volumes. Pairwise comparisons were performed for significant main effects by conducting nonparametric Mann–Whitney tests. Bonferroni correction was applied (*P*_corr_ = *P* × 3) for group comparisons and we report adjusted *P*_corr_ values unless indicated otherwise. The critical *P*_corr_ value was set at 0.05.

For global brain measurements, the Benjamini–Hochberg false discovery rate (FDR) correction of 0.05% was applied to correct for multiple testing. Both uncorrected and FDR-corrected *P* values are reported. No correction was performed for subcortical and cortical analyses as they were of exploratory nature.

For exploratory analyses, volume measures were corrected for total intracranial volume (TIV) by dividing each value by the individual’s TIV value, which resulted in values between 0 and 1, reflecting relative size of the particular structure in relation to the TIV. TIV was calculated as the sum of total cerebral grey and white matter volumes, lateral, third and fourth ventricles, choroid plexus, vessels, cerebellar grey and white matter, and surface CSF.

## Results

Clinical data are presented in Table 1. No significant differences were found in age, handedness, and gender distribution among groups, and no differences in IQ scores and age at start of growth hormone (GH) treatment. Only two children in the mUPD group were diagnosed with ASD. None of the children had a history of treatment with psychotropic medication.

**Table 1:** Demographic data of the participants

	**PWS**		**Control**	***P *** **value**
	**DEL**	**mUPD**		
Age (years)	12.3 (3.2)	10.6 (2.5)	11.7 (2.7)	0.40
Age range (years)	6.7 to 17.0	6.8 to 13.1	7.1 to 15.8	
Head circumference SDS	−0.37 (0.84)	0.8 (1.2)	n/a	0.11^a^
Sample size (n)	11	9	11	0.26
Male	5	4	8	0.60
Female	6	5	3	0.50
Handedness **(n)**				0.25
Left	1	6	1	
Right	10	1	10	
Ambidextrous	0	2	0	
Age at start of GH treatment (years)	5.8 (3.0)	4.4 (1.8)		0.37
Total IQ score	69.5 (16.0)	67.2 (14.5)		0.82
Verbal IQ	4.7 (3.4)	4.0 (2.6)		0.66
Performance IQ	4.7 (2.8)	4.2 (2.9)		0.60
Psychiatric history	0	2^b^		
Use of psychotropic medication	0	0		

Data expressed as mean (SD) or number. No significant differences were found in either age or gender distribution across groups. ^a^Values of DEL and mUPD were compared to 0 SDS; ^b^two children diagnosed with ASD prior to MRI scan. *DEL*, deletion; *GH*, growth hormone; *mUPD*, maternal uniparental disomy; n/a, not applicable; *PWS*, Prader–Willi syndrome; *SD*, standard deviation; *SDS*, standard deviation score.

### Global brain measures

The results of global measures are presented in Table 2.

**Table 2:** Global brain volumes in children with PWS and healthy controls

	**PWS**				**Control**		***P *** **value**	***P***_**corr **_**value**	***P***_**corr **_**value**	***P***_**corr **_**value**
							**Between groups**	**DEL versus control**	**mUPD versus control**	**mUPD versus DEL**
							**(FDR-corrected)**			
	**DEL**		**mUPD**							
	**Mean**	**SD**	**Mean**	**SD**	**Mean**	**SD**				
TIV (mm^3^)	1,234,546	107,087	133,1869	128,966	1,410,393	98,231	0.007 (0.02)	0.005	ns	ns
Total GM (mm^3^)	684,884	59,560	754,819	74,391	788,263	67,474	0.008 (0.02)	0.007	ns	0.118
Cortical GM (mm^3^)	511,270	53,409	563,276	65,484	588,374	60,604	0.026 (0.047)	0.026	ns	ns
Subcortical GM (mm^3^)	173,615	12,739	191,543	11,495	199,890	2,525	<0.001 (0.001)	<0.0001	ns	0.036
Total WM (mm^3^)	408,399	45,050	413,740	60,537	462,809	38,073	0.034 (0.053)	0.061	0.098	ns
Total CC (mm^3^)	3,053	345	2,784	533	3,010	342	ns (ns)	ns	ns	ns
Brainstem (mm^3^)	16,207	1,378	17,384	1,511	20,493	1,858	<0.001 (0.001)	<0.0001	0.016	ns
Ventricles (mm^3^)										
Lateral (mm^3^)	14,318	11,606	20,598	8,781	9,244	610	0.001 (0.004)	ns	0.006	ns
Third (mm^3^)	932	433	1,091	275	868	190	ns (ns)	ns	ns	ns
Fourth (mm^3^)	1,986	495	1,952	394	1,853	455	ns (ns)	ns	ns	ns
Cerebellum (mm^3^)	124,737	11,456	141,378	9,644	146,092	11,022	0.001 (0.004)	0.001	ns	0.042
Surface CSF (mm^3^)	1,199	395	1,469	253	1,077	255	0.027 (0.047)	ns	0.029	0.133
Mean cortical thickness (mm)	2.77	0.104	2.89	0.185	2.8	0.106	0.066 (0.08)			
Mean pial cortical area (mm^2^)	221,117	22,519	232,867	20,785	249,491	23,373	0.05 (0.07)			

Both uncorrected and FDR-corrected *P* values are reported. *P*_corr_ values that did not survive the Bonferroni correction are included. CC, corpus callosum; CSF, cerebrospinal fluid; *DEL*, deletion; *FDR*, false discovery rate; *GM*, grey matter; *mUPD*, maternal uniparental disomy; ns, not significant; *PWS*, Prader–Willi syndrome; *SD*, standard deviation; *TIV*, total intracranial volume; *WM*, white matter.

### DEL versus healthy controls

Children with DEL had a significantly smaller TIV (*P*_corr_ <0.01) when compared with healthy controls. They also had a significantly smaller total grey matter volume (*P*_corr_ <0.01), both cortical (*P*_corr_ <0.05) and subcortical (*P*_corr_ <0.01), total white matter volume (*P* = 0.02, not significant after Bonferroni correction), brainstem (*P*_corr_ <0.01), and cerebellum (*P*_corr_ <0.01) (Table 2). No differences were found in corpus callosum volume, lateral, third and fourth ventricles, and surface CSF compared with healthy controls.

After correction for TIV, the ratios of grey and white matter volume, brainstem, cerebellum, and lateral, third, and fourth ventricles to TIV were not significant between children with DEL and controls, which suggests that although overall smaller, the brain had developed proportionally (Table 3). However, the corpus callosum volume, corrected for TIV, was relatively larger (*P*_corr_ <0.05).

**Table 3:** Focal cortical grey matter volumes in children with PWS and healthy controls

	**PWS**				**Control**		***P *** **value between groups**	***P***_**corr **_**value DEL versus control**	***P***_**corr **_**value mUPD versus control**	***P***_**corr **_**value mUPD versus DEL**
	**DEL**		**mUPD**							
	**Mean**	**SD**	**Mean**	**SD**	**Mean**	**SD**				
Left entorhinal	0.00153	0.00025	0.00123	0.00039	0.00148	0.00033	0.028	ns	0.127	0.031
Left banks of STS	0.00237	0.00027	0.00217	0.00053	0.00204	0.00032	0.041	0.06	ns	0.142
Right superior parietal	0.01359	0.00127	0.01363	0.00142	0.01163	0.00099	0.002	0.006	0.006	ns
Right lingual	0.00621	0.00072	0.00532	0.00087	0.00584	0.00059	0.032	ns	ns	0.027
Right parahippocampal	0.00162	0.00021	0.00154	0.0003	0.00194	0.00036	0.014	0.11	0.016	ns
Right banks of STS	0.00201	0.00026	0.00237	0.00033	0.00202	0.00045	0.032	ns	0.045	0.091

All measures presented in table are corrected for TIV. *P*_corr_ values that did not survive the Bonferroni correction are included. DEL, deletion; mUPD, maternal uniparental disomy; ns, not significant; PWS, Prader–Willi syndrome; SD, standard deviation; STS, superior temporal sulcus; TIV, total intracranial volume.

### mUPD versus healthy controls

**Figure 1:**
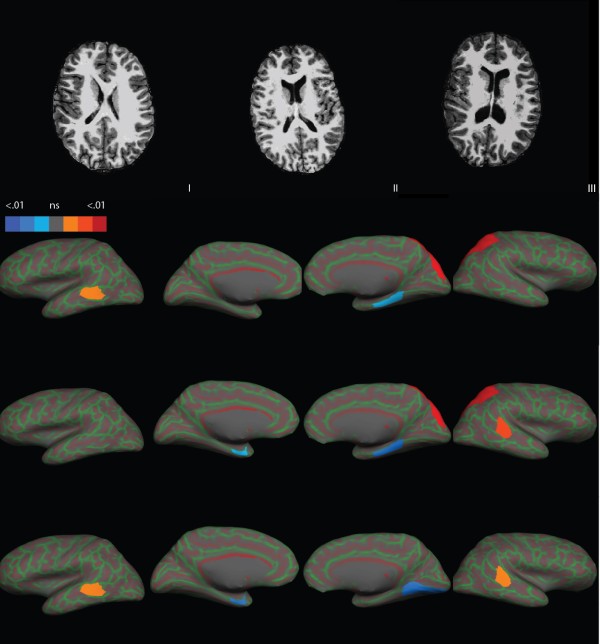
**Ventricular enlargement and cortical focal grey matter volumes in children with PWS and healthy controls.** Top row: ventricular enlargement in children with PWS. **(I)** Healthy control; **(II)** child with DEL; and **(III)** child with mUPD. All three presented children were of the same age and gender. Lower three rows: cortical focal measures in children with PWS and healthy controls. Cortical surface is presented inflated with curvature overlay (in green and red shades). Second row: children with DEL compared to healthy controls; third row: children with mUPD compared to healthy controls; last row: children with mUPD compared to children with DEL. All the views are in sagittal plane. Columns (from left to right): left lateral, left medial, right medial, and right lateral. DEL, deletion; mUPD, maternal uniparental disomy; PWS, Prader–Willi syndrome.

No difference was found in TIV between children with mUPD and healthy controls. They also had normal total grey matter (cortical and subcortical) volumes compared with healthy controls. Total white matter volume was smaller (*P* = 0.03, not significant after Bonferroni correction). Children with mUPD also had a smaller brainstem volume (*P*_corr_ <0.05), enlarged lateral ventricles (*P*_corr_ <0.01), and larger surface CSF (*P*_corr_ <0.05) (see also Figure 1). No differences were found in cerebellum and corpus callosum volume or third and fourth ventricles (Table 2).

After correction for TIV, a significantly disproportionate increase in the volume of lateral ventricles (*P*_corr_ <0.01) and surface CSF (*P*_corr_ <0.05) was found (Additional file 1: Table S1).

### DEL versus mUPD

Children with DEL had a smaller total grey matter volume (*P* = 0.04, not significant after Bonferroni correction), subcortical grey matter volume (*P*_corr_ <0.05), and cerebellum (*P*_corr_ <0.05) compared with children with mUPD (Table 2).

After correction for TIV, children with DEL had significantly larger corpus callosum than children with mUPD (*P*_corr_ <0.05) (Additional file 1: Table S1).

### Exploratory focal measures

#### Cortical grey matter volumes

Cortical grey matter volumes, corrected for TIV, are presented in Table 3 and Figure 1. We report only significant results. The non-significant findings can be provided upon request.

### DEL versus healthy controls

Children with DEL had a significantly larger volume of right superior parietal lobe (*P*_corr_ <0.01), larger volume of banks of left superior temporal sulcus (*P* = 0.02, not significant after Bonferroni correction), and smaller volume of right parahippocampal gyrus (*P* = 0.037, not significant after Bonferroni correction) compared with healthy controls.

### mUPD versus healthy controls

Children with mUPD had smaller grey matter volumes in right parahippocampal gyrus (*P*_corr_ <0.05) and left entorhinal (*P* = 0.04, not significant after Bonferroni correction) compared with healthy controls. Further, children with mUPD had significantly larger right superior parietal lobe (*P*_corr_ <0.01) and banks of right superior sulcus (*P*_corr_ <0.05).

### DEL versus mUPD

Children with mUPD had smaller cortical grey matter volumes in right lingual gyrus (*P*_corr_ <0.05), left entorhinal (*P*_corr_ <0.05), and banks of left and right superior temporal sulcus (*P* = 0.047 and *P* = 0.03, respectively, not significant after Bonferroni correction) when compared to children with DEL, but not from those in healthy controls.

### Cortical white matter volumes

**Figure 2:**
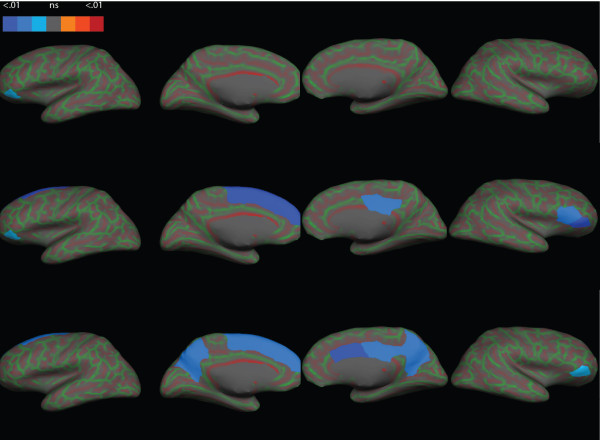
**Focal white matter volumes in children with PWS and healthy controls.** Cortical surface is presented inflated with curvature overlay (in green and red shades). Top row: children with DEL compared to healthy controls; middle row: children with mUPD compared to healthy controls; last row: children with mUPD compared to children with DEL. All the views are in sagittal plane. Columns (from left to right): left lateral, left medial, right medial, and right lateral. DEL, deletion; mUPD, maternal uniparental disomy; PWS, Prader–Willi syndrome.

Cortical white matter volumes, corrected for TIV, are presented in Table 4 and Figure 2. We report only significant findings. The non-significant findings can be provided upon request.

**Table 4:** Focal cortical white matter volumes in children with PWS and healthy controls

	**PWS**				**Control**		***P *** **value between groups**	***P***_**corr **_**value DEL versus control**	***P***_**corr **_**value mUPD versus control**	***P***_**corr **_**value mUPD versus DEL**
	**DEL**		**mUPD**							
	**Mean**	**SD**	**Mean**	**SD**	**Mean**	**SD**				
Left pars orbitalis	0.00057	0.00008	0.00055	0.00008	0.00064	0.00006	0.033	0.09	0.06	ns
Left superior frontal	0.01242	0.0012	0.01101	0.00074	0.01284	0.00032	0.002	ns	0.001	0.026
Left precuneus	0.00679	0.00098	0.00566	0.00096	0.00652	0.00065	0.025	ns	ns	0.021
Right pars orbitalis	0.0008	0.00013	0.00070	0.00008	0.00088	0.00011	0.005	ns	0.003	0.127
Right pars triangularis	0.00217	0.00031	0.00190	0.00035	0.0023	0.00038	0.014	ns	0.043	ns
Right caudal anterior cingulate	0.00235	0.00024	0.00237	0.00015	0.00219	0.00034	0.011	ns	ns	0.008
Right posterior cingulate	0.00319	0.00025	0.00198	0.00022	0.00314	0.00026	0.008	ns	0.044	0.010
Right precuneus	0.00736	0.00125	0.00282	0.00089	0.00678	0.00079	0.042	ns	ns	0.036

All measures presented in table are corrected for TIV. *P*_corr_ values that did not survive the Bonferroni correction are included. *DEL*, deletion; *mUPD*, maternal uniparental disomy; ns, not significant; *PWS*, Prader–Willi syndrome; *SD*, standard deviation; *TIV*, total intracranial volume.

### DEL versus healthy controls

Children with DEL had smaller white matter volume of left pars orbitalis (*P* = 0.03, not significant after Bonferroni correction) compared with healthy controls. No other differences were found.

### mUPD versus healthy controls

Children with mUPD had smaller white matter volumes in left superior frontal gyrus (*P*_corr_ <0.01), left pars orbitalis (*P* = 0.02, not significant after Bonferroni correction), right pars triangularis (*P*_corr_ <0.05), right pars orbitalis (*P*_corr_ <0.01), and right posterior cingulate (*P*_corr_ <0.05) compared with healthy controls.

### DEL versus mUPD

Children with mUPD had smaller white matter volumes in bilateral precuneus (*P*_corr_ <0.05) and right caudal anterior cingulate cortex (*P*_corr_ <0.01) when compared to children with DEL. Further, these children had smaller right pars orbitalis (*P* = 0.042, not significant after Bonferroni correction), left superior frontal gyrus (*P*_corr_ <0.05), and right posterior cingulate cortex (*P*_corr_ <0.01) compared with children with DEL and healthy controls.

### Subcortical grey matter structures

Subcortical grey matter volumes, corrected for TIV, are presented in Additional file 2: Table S2.

### DEL versus healthy controls

Children with DEL had enlarged volumes of left basal ganglia (*P*_corr_ <0.05) compared with healthy controls. No differences were found in bilateral limbic area, right basal ganglia, and bilateral thalamus.

### mUPD versus healthy controls

Children with mUPD had larger left basal ganglia (*P* = 0.026, not significant after Bonferroni correction) when compared with healthy controls. No other differences were observed.

### DEL versus mUPD

No differences were found in volumes of subcortical grey volumes between children with DEL and mUPD.

## Discussion

Our study is the first to investigate brain morphological differences in children with PWS using high resolution MRI techniques, and the first study to investigate differences between children with DEL and mUPD. We found that children with both DEL and mUPD had smaller brainstem volumes, and a trend towards smaller white matter volume and total cortical pial surface area compared with healthy controls, indicating early deviations in prenatal brain development in children with PWS. However, there were also differences between children with DEL and those with mUPD. Children with DEL had an overall smaller, but proportionately developed brain and normal cortical thickness, while children with mUPD had a significantly increased surface CSF and pronounced enlargement of the lateral ventricles compared to healthy controls. Further, exploratory cortical focal analyses revealed a significantly smaller white matter volume in children with mUPD in areas implicated in default mode network (DMN) and cognitive decision-making. Our results suggest that although both PWS groups show global brain deficits, children with mUPD are more severely affected.

Prenatal brain development is a well-orchestrated series of events consisting of neuronal proliferation and migration, establishment of synaptic connections, myelination, and elimination of ineffective synapses (pruning) [31]. Different regions of the brain develop at different time points and pace, which in turn provides different time windows for vulnerability to perturbations, but also for effective intervention. For instance, development of brainstem is finalized during the first trimester, while development of cerebellum is initialized during the first trimester, but extends into the first year after birth [31]. Myelination is most vulnerable to insults during the late gestational period, but it continues after birth as well and extends through adolescence and into early adulthood [32]. By investigating both global and local neuroanatomical measures in children with PWS, inferences can therefore be drawn about a plausible course of neurodevelopment and the phenotype of PWS.

Both children with DEL and mUPD had smaller brainstem volume. The brainstem is responsible for several basal bodily functions, such as pain perception, respiratory regulation [33], and sleep cycle [34], all of which have been reported to be impaired in PWS. Central sleep apneas are common in individuals with PWS and pose an increased mortality risk at a young age [35]. A higher pain threshold has been reported in children with PWS [2], as well as disturbed sleep cycle and rapid eye movement (REM) sleep phase abnormalities [36], supporting our findings of possible brainstem dysfunction in PWS. Mice lacking necdin (in PWS locus) had smaller medullar nuclei and a disturbed respiratory rhythmogenesis [37], further suggesting that PWS region is involved in the neurodevelopment of the brainstem and the respiratory problems in patients with PWS.

Regardless of the genetic subtype, children with PWS had a trend towards smaller white matter volume, indicating reduced structural connectivity or aberrant myelination in children with PWS. Children with developmental delay showed delayed myelination [38]. The smaller white matter volume in children with PWS may therefore reflect delay in brain maturation and might underlie cognitive deficits in these children. Important to note is that several endocrine factors, namely insulin-like growth factor I (IGF-I) and thyroid hormones (T3 and T4), which are important neurochemicals involving brain and axonal growth and myelination [39,40], are impaired in children with PWS, unless treated. IGF-I levels are very low in most children with PWS prior to GH treatment [17]. Low free T4 levels were reported in children with PWS as well [41]. It is therefore plausible that lower levels of thyroid hormones and IGF-I had adverse effects on brain growth and myelination.

Children with mUPD had enlarged ventricles, together with increased surface CSF volume. In adult patients with PWS, enlarged ventricles have been reported [16]. Our results suggest that ventricular enlargement occurs early in life in individuals with PWS. We do not know when during neurodevelopment the enlargement of the ventricles took place. Lateral ventricles are large at the 17th gestational week (GW), and decrease in size between 18th and 24th GW, due to multiple factors, including the increasing thickness of the brain parenchyma, cortical gyrification, and the formation of the basal ganglia [42]. In the current study, the basal ganglia were enlarged relative to the intracranial volume, which may indicate that ventricular enlargement did not occur at the cost of the basal ganglia, but more likely at the cost of the cortical volumes. Enlarged ventricles, together with increased surface CSF, probably due to the widening of the sulci, indicate disturbances in early prenatal gyrification, dendritic arborization, or postnatal neuronal atrophy in these children. Enlarged ventricles are widely reported in patients with schizophrenia, both chronic [43] and first onset medication-naive patients with schizophrenia [44], and their first-degree unaffected relatives [45]. Furthermore, enlarged lateral ventricles were also found in young children with a 22q11.2 deletion who are at very high risk of schizophrenia [46] and in adolescents with a psychotic bipolar disorder [47]. Given that children with mUPD have an elevated risk of psychotic illness, our findings suggest that ventricular enlargement may be part of a predisposition for psychotic disease.

Children with DEL showed less enlargement of the lateral ventricles and no increase in surface CSF, suggesting that brain atrophy is less pronounced in these children compared to children with mUPD. Since ventricular enlargement has been reported in adults with DEL [16], our findings suggest that children with DEL are likely to develop ventricular enlargement later in life compared to children with mUPD.

Both children with DEL and mUPD showed a trend towards smaller cortical surface area, indicating impaired prenatal brain growth [31]. The development of cortical surface area is determined by the symmetric cell division in the neural tube during the first 6 GW, and by the pronounced growth and gyrification that occurs during the third trimester [42]. Environmental insults during the first 6 GW usually results in drastic reductions of cortical surface area [31]. It is therefore plausible that the observed mild reduction in cortical surface area may be the result of deviations in the gyrification processes during the third trimester.

Interestingly, children with DEL show a different pattern compared to children with mUPD. Smaller cortical and subcortical grey matter, brainstem, and cerebellum volumes were found, but cortical thickness was normal compared to healthy controls. The early development of cortical thickness is determined primarily through neuronal migration, which takes place between 6th and 24th GW [48]. Furthermore, from the perspective of phylogeny, cortical thickness is much more preserved compared to measures, such as brain volume and surface area [31]. Thus, normal cortical thickness may show that the genetic mechanisms involved in neuronal migration are not altered in children with DEL.

In contrast, children with mUPD showed a trend towards increased cortical thickness, which may indicate alterations in neuronal migration or impaired elimination of ineffective synapses (pruning). Increased cortical thickness has been reported in children with autism [49]. Knowing that ASD traits are very common in children with mUPD, impaired pruning might therefore affect integrative processing, complex executive, and social functions [49], and might underlie ASD symptoms in children with mUPD.

Holland *et al*. [50] proposed that developmental arrest might underlie the core PWS phenotype. In our study, both children with DEL and mUPD showed signs of impaired brain growth, however it was more pronounced in children with DEL. Furthermore, while children with DEL show signs of arrested, but not necessarily deviant, brain development, children with mUPD showed a plausible divergence in the neurodevelopmental trajectory. However, longitudinal studies are necessary to confirm the differences in neurodevelopmental trajectories between children with DEL and mUPD. An important genetic difference between individuals with DEL and mUPD is the overexpression of maternally imprinted *UBE3A* gene in brains of patients with mUPD [51]. *UBE3A* plays an important role in dendritic tree formation [52], and both knockout and overexpression of the *UBE3A* gene results in reduced growth and branching of the dendrites [52]. *UBE3A* regulates synapse development and is highly expressed during a novel learning situation [53]. Overexpression of *UBE3A* in mUPD and resulting failure to develop new synapses might be underlying the observed diversion, and possibly the cortical atrophy and ventricular enlargement in children with mUPD.

Exploratory cortical focal analyses revealed smaller white matter volume in the right caudal anterior and posterior cingulate cortex, left superior frontal gyrus, and bilateral precuneus in children with mUPD compared to those with DEL and to healthy controls. These areas are associated with cognitive control, moral decision-making, and emotion cognition [54], and are implicated in DMN [55,56]. The DMN is a functional brain network that is activated in the absence of cognitive tasks [56], and is thought to reflect self-oriented and social cognitive processes [57]. DMN dysfunction is associated with multiple brain disorders, such as attention deficit and hyperactivity disorder, autism, and schizophrenia [56,58-60]. Interestingly, electrophysiological correlates of decision-making was found diminished in adults with mUPD, but not in those with DEL [61], and individuals with mUPD had more autistic-like symptoms on the social interaction scale [62]. Impairment of task-switching was found in individuals with PWS, although genetic subtypes were not reported [63]. While this should be confirmed by functional imaging studies, it is plausible that aberrant connections within these brain areas underlie social cognitive decision-making impairment in individuals with mUPD. Further, white matter volume in right pars triangularis and bilateral pars opercularis (inferior frontal gyrus (IFG)) was smaller in children with mUPD. Interestingly, lower functional connectivity in the IFG has been described in patients with schizophrenia and in individuals at ultra-high risk of developing psychosis [64], suggesting that IFG might be involved in the etiology of psychotic illness.

These results are limited by the small sample size, thus generalization to broader PWS population should be undertaken with great caution. Further, the control group consists of age- and gender-matched healthy siblings. A possible concern of recruiting siblings as the control group is that volumes of most brain structures are heritable [65]. However, as PWS occurs due to a *de novo* genetic event during conception, we assume that unaffected siblings are representative of a random sample of the general population. The great advantage of having siblings as the control group is that the effects of possible environmental and hereditary factors on brain development are greatly reduced, and that the observed significant differences are more likely PWS-specific.

All children with PWS were treated with GH (1 mg/m^2^ per day) at the time of the study; therefore, our findings are confounded by GH treatment. However, as it is known that GH and IGF-I increase brain growth, myelination, and has neuroprotective properties [39] we could speculate that if the GH treatment had any effect of the brain, it would have a positive effect in terms of brain normalization.

## Conclusions

Our findings provide preliminary insights into the brain anatomy of children with PWS. All children with PWS showed impaired brain growth. Children with mUPD showed signs of early brain atrophy and a trend towards increased cortical thickness. In contrast, children with DEL showed signs of fundamentally arrested, although not deviant brain development. The findings in children with mUPD are reminiscent of those in schizophrenia or autism, which confirms the clinical data of increased risk of ASD and psychotic illness in individuals with mUPD. The reported brain abnormalities are likely to precede psychiatric illness as none of the children were under psychiatrist treatment at the time of the study. Our results suggest that children with mUPD have a fundamentally different brain structure and divergent developmental trajectories compared with children with DEL and healthy controls.

## Abbreviations

ASD: Autism spectrum disorders; CNS: Central nervous system; CSF: Cerebrospinal fluid; CNVs: Copy-number variations; CC: Corpus callosum; DMN: Default mode network; DEL: Deletion; TE: Echo time; FDR: False discovery rate; GWAS: Genome-wide association study; GW: Gestational week; GM: Grey matter; GH: Growth hormone; IFG: Inferior frontal gyrus; IGF-I: Insulin-like growth factor I; IQ: Intelligence quotient; IR FSPGR: Inversion recovery fast spoiled gradient recalled; TI: Inversion time; MRI: Magnetic resonance imaging; mUPD: Maternal uniparental disomy; NEX: Number of excitations; OCD: Obsessive-compulsive disorder; PWS: Prader–Willi syndrome; REM: Rapid eye movement; TR: Repetition time; SD: Standard deviation; SDS: Standard deviation score; STS: Superior temporal sulcus; TIV: Total intracranial volume; WISC-R: Wechsler Intelligence Scale for Children-Revised; WM: white matter.

## Competing interests

The authors declare that they have no competing interests.

## Authors’ contributions

AL, ACHK and AVDL designed the study. AL collected and analyzed the data and wrote the manuscript. TW contributed to the analysis and interpretation of the data as well as writing of the manuscript. TW and MS helped with technical aspects of the MRI acquisition, data processing and analysis. ACHK is a principal investigator and supervisor of the project, and contributed to data analysis and writing of the manuscript. All authors read and approved the final manuscript.

## Supplementary Material

Additional file 1: Table S1 Whole brain measures in children with PWS and healthy controls. Click here for file

Additional file 2: Table S2 Subcortical structure measures in children with PWS and healthy controls. Click here for file
