# Supplementary figures and images for: Divergent structural brain abnormalities between different genetic subtypes of children with Prader–Willi syndrome
Source: J Neurodev Disord. 2013 Oct 22;5(1):31. doi: 10.1186/1866-1955-5-31 (PMC4015928; doi:10.1186/1866-1955-5-31)

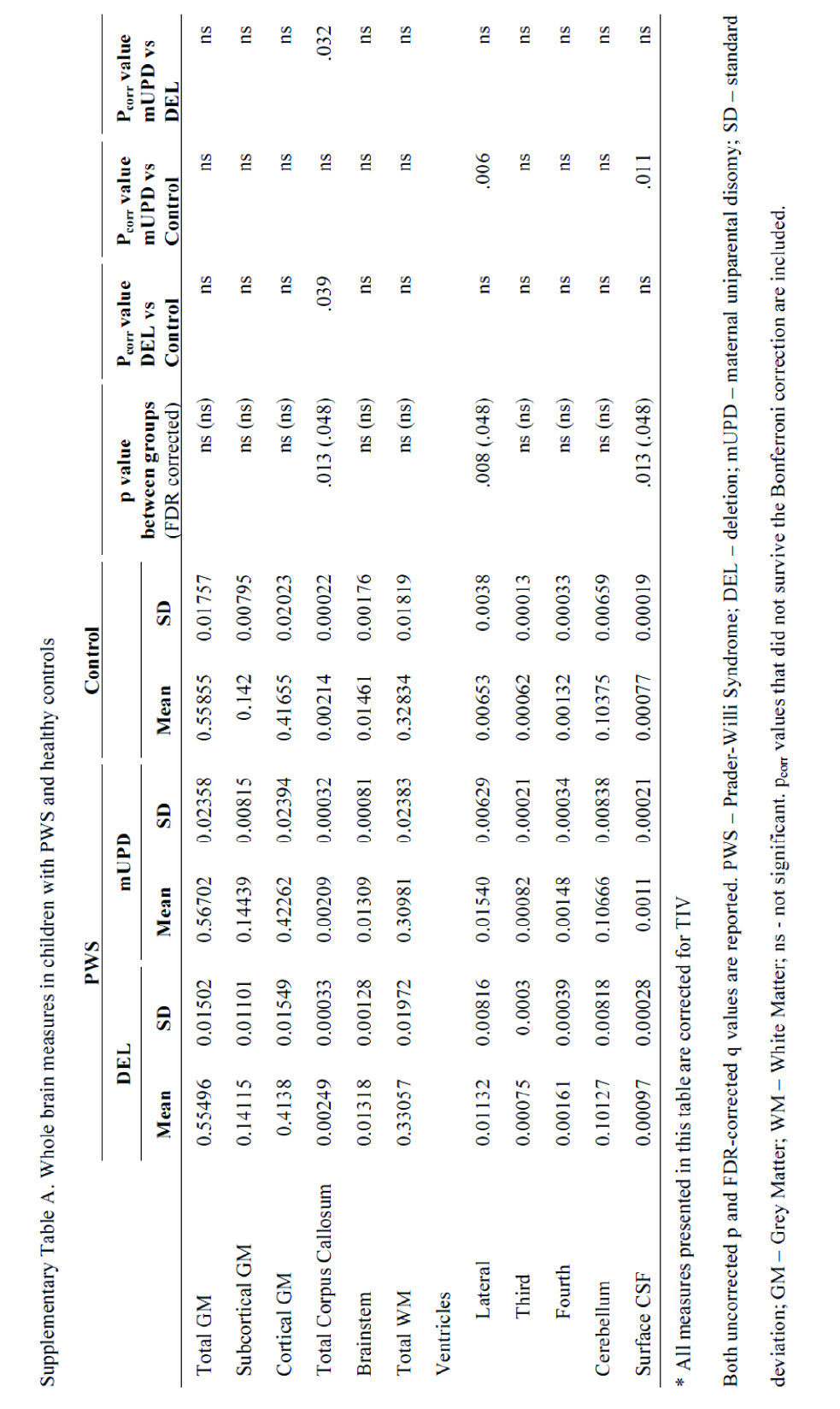

Supplement: Additional file 1: Table S1 — Whole brain measures in children with PWS and healthy controls. [file 1866-1955-5-31-S1.tiff]

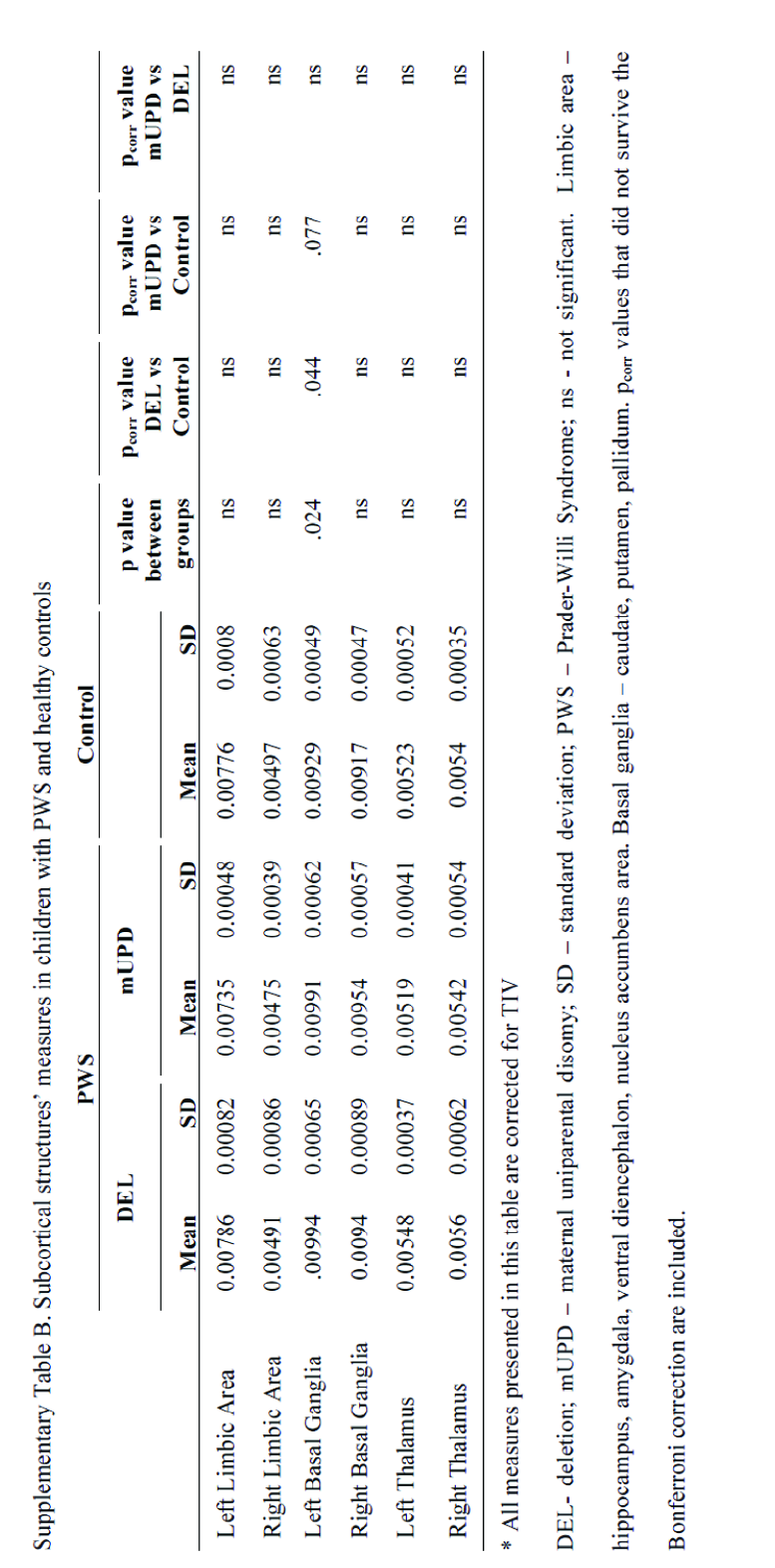

Supplement: Additional file 2: Table S2 — Subcortical structure measures in children with PWS and healthy controls. [file 1866-1955-5-31-S2.tiff]
